# Spontaneous Re-eruption of Severely Intruded Immature Permanent Maxillary Central Incisors and Orthodontic Treatment

**DOI:** 10.7759/cureus.117946

**Published:** 2026-10-14

**Authors:** Nuran Gokdogan, Volkan Ciftci

**Affiliations:** 1 Department of Pedodontics and Preventive Dentistry, Cukurova University, Adana, TUR

**Keywords:** cvek’s classification, immature permanent tooth, intrusive luxation, removable appliance, spontaneous re-eruption

## Abstract

Intrusive luxation is a serious traumatic dental injury that causes significant damage to the periodontium, alveolar bone, and pulp. Severe intrusions are generally associated with poor prognoses and a high risk of complications. This report presents the successful spontaneous re-eruption of severely intruded immature permanent maxillary central incisors. An eight-year-old boy was referred with severe intrusion of the maxillary right and left central incisors to the gingival level, with an intrusion depth of approximately 7 mm-8 mm. In accordance with the International Association of Dental Traumatology (IADT) guidelines, a “wait-and-watch” approach was adopted. The teeth completely re-erupted spontaneously within 14 months of treatment. At the 36-month follow-up, after allowing for root maturation and periodontal stabilization, a removable orthodontic appliance was used to correct the palatal tipping of the right maxillary central incisor, a sequela of intrusive luxation. Over the 58-month follow-up period, no signs of pulp necrosis, root resorption, or ankylosis were observed. This case report supports the notion that severely intruded immature permanent incisors can undergo successful spontaneous re-eruption. Furthermore, this finding is consistent with emerging evidence suggesting that Cvek’s classification may serve as a useful prognostic indicator of the potential for spontaneous re-eruption in immature permanent teeth.

## Introduction

Traumatic dental injuries (TDIs) are highly prevalent in children and most frequently result from falls during active play, sports, and school activities. Intrusive luxation is characterized by axial displacement of a tooth into the alveolar socket. This type of injury is most prevalent in boys aged six to 12 years, with the maxillary central incisors being the most frequently affected, and its incidence ranges from 0.5% to 1.9% of all permanent TDIs [1-5].

The impact forces during intrusive luxation disrupt periodontal ligament (PDL) fibers in the alveolar socket and can damage socket walls. Furthermore, the displacement of the plaque-covered surface of the crown into the periodontium increases the risk of infection [2]. In teeth with incomplete root development, there is a risk of apical pulp tissue severance and introduction of socket bone into the open apex [6]. These factors predispose teeth to complications such as pulp necrosis, external inflammatory resorption, replacement resorption (ankylosis), pulp canal obliteration, and marginal bone loss. Replacement root resorption is considered the most severe complication because of the lack of definitive treatment [5,7] and its potential to lead to premature tooth loss and subsequent malocclusion in young patients [8].

The management of intrusive luxation remains challenging because of its relatively low incidence, which has led to various treatment protocols [3,7,9]. Current strategies are primarily influenced by the stage of root development (maturation), the severity of intrusion, and the patient’s age [10]. The International Association of Dental Traumatology (IADT) recommends allowing immature teeth to re-erupt spontaneously without immediate intervention [11,12]. Immature teeth with open apices have better prognoses and lower complication rates because of their potential for revascularization. When spontaneous re-eruption fails, or when partial eruption, pulp necrosis, or replacement resorption occurs, active interventions such as orthodontic extrusion, surgical repositioning, and complex endodontic procedures may be required [13].

Some studies have highlighted a strong inverse relationship between the depth of intrusion and the likelihood of spontaneous re-eruption, with severe intrusions (>7 mm) often associated with a poor prognosis for re-eruption, even in immature teeth [14,15]. Furthermore, a recent study demonstrated that the probability of spontaneous re-eruption significantly decreased as the root matured. Long-term outcomes for severely intruded (>7 mm) immature teeth at Cvek Stage 1 remain underreported, particularly regarding post-eruption orthodontic management [16]. This case report addresses this gap by documenting a 58-month follow-up, including successful spontaneous re-eruption and subsequent correction of post-traumatic malposition using a removable appliance.

Written informed consent was obtained from the child’s legal guardian (sister) for the diagnostic and therapeutic procedures and publication of this case report, including all accompanying clinical photographs and radiographs.

## Case presentation

An eight-year-old boy was referred to Cukurova University, Faculty of Dentistry, Department of Pediatric Dentistry, in September 2021, with a complaint of pain and bleeding in the maxilla following a fall at school on the same day. There was no history of loss of consciousness or cerebrospinal fluid leakage from the nose or ears. Intraoral examination revealed gingival hemorrhage, avulsion of the deciduous right lateral incisor, and severe intrusion of the maxillary right and left central incisors to the gingival level, with an intrusion depth of approximately 7 mm-8 mm (Figure 1).

**Figure 1:**
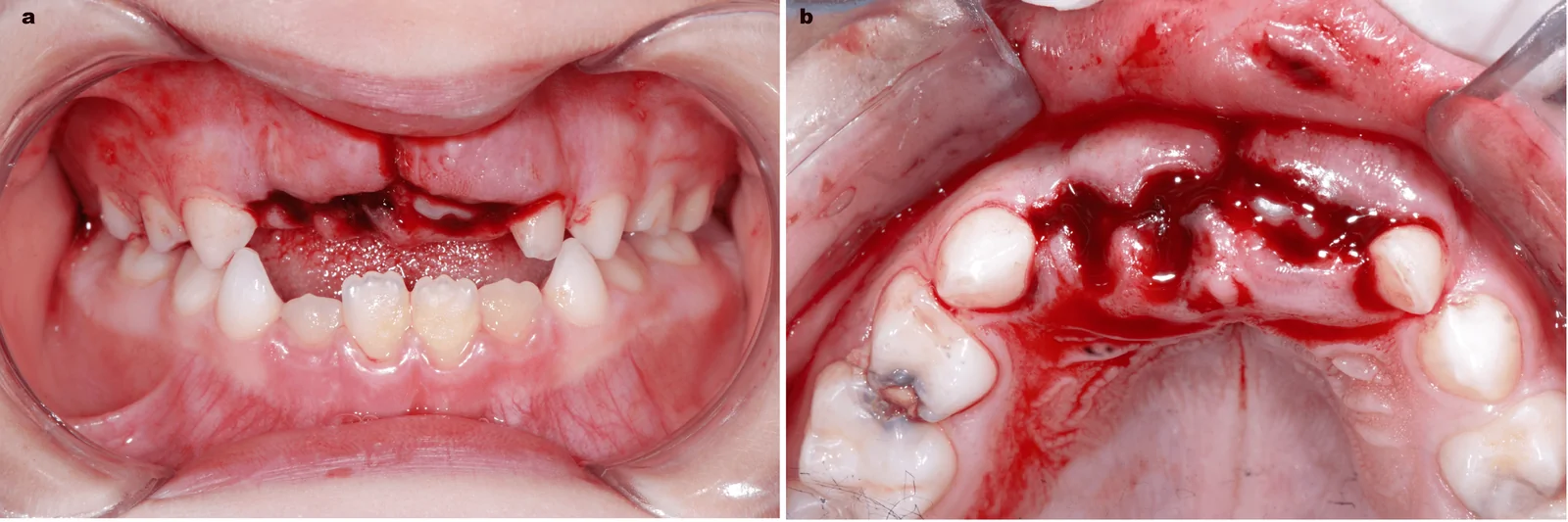
Initial intraoral views: (a) frontal and (b) occlusal views showing gingival hemorrhage, severe intrusion (7-8 mm) of the maxillary right and left central incisors to the gingival level, and avulsion of the maxillary right deciduous lateral incisor

Orthopantomogram and intraoral periapical radiographs (Figure 2) revealed immature root development, classified as Cvek Stage 1 (an open, funnel-shaped apex with a root length less than the crown height). In accordance with the 2020 IADT guidelines, a conservative “wait-and-watch” approach was adopted to monitor spontaneous re-eruption, and the patient was closely monitored over a four-week period [12]. Although the 2020 IADT guidelines do not routinely recommend systemic antibiotics for isolated intrusion injuries, a five-day course of systemic antibiotics (amoxicillin/clavulanic acid, 625 mg) was prescribed in this case. This proactive decision was clinically justified by the heavy contamination of the severely crushed gingival tissues following the outdoor fall and the presence of an open, bleeding socket resulting from the concomitant avulsion of the deciduous lateral incisor, both of which increased the risk of secondary infection. Additionally, analgesics (paracetamol) and 0.2% chlorhexidine gluconate mouthwash were prescribed to support periodontal healing and plaque control, along with oral hygiene education.

**Figure 2:**
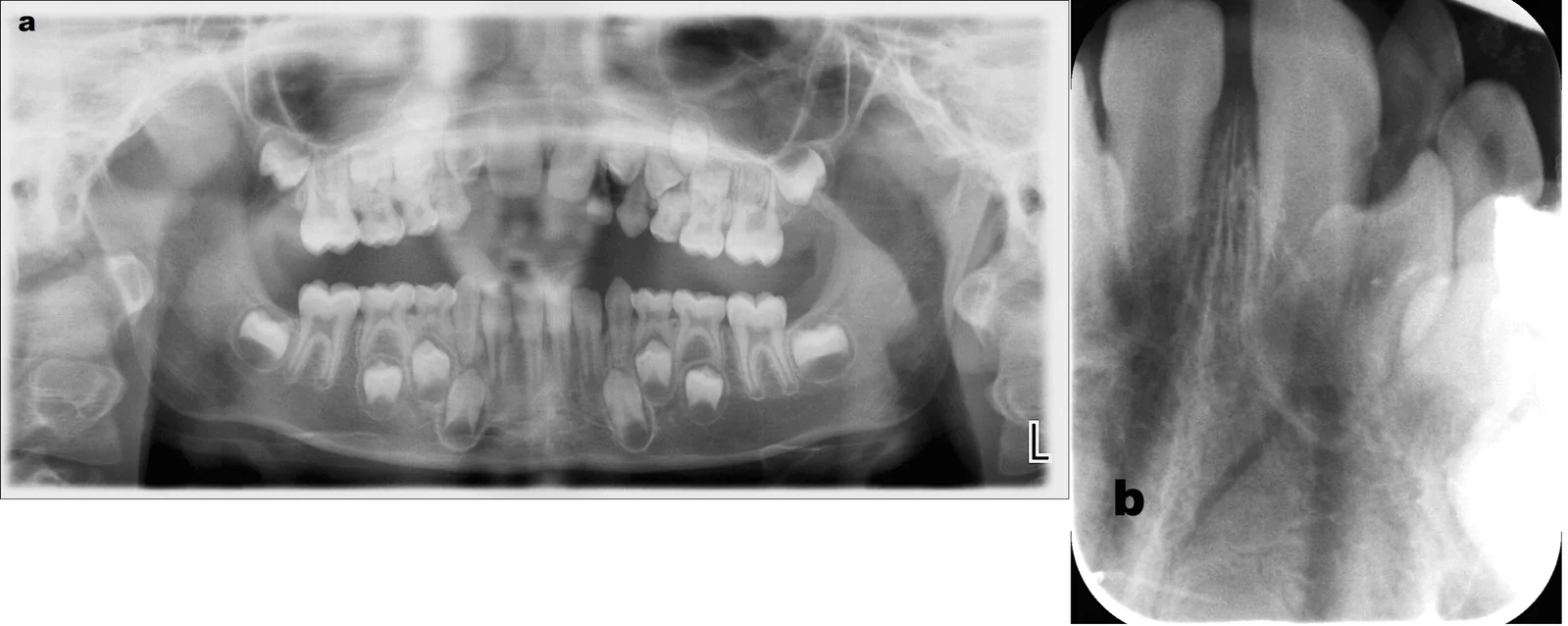
Initial radiographic evaluation: (a) orthopantomogram and (b) periapical radiograph confirming severe intrusion of the maxillary central incisors and showing immature root development with open, funnel-shaped apices

During the observation period, follow-up radiographs were strictly selected based on as low as diagnostically acceptable, indication-based, and person-oriented (ALADAIP) radiation safety principles. Intraoral periapical radiographs were limited to specific diagnostic indications: at four weeks to evaluate initial periodontal healing and rule out early inflammatory resorption; at three months to monitor vertical eruptive progress; at 14 months, following complete eruption, to assess root lengthening; and at 40 months, before orthodontic force application, to assess PDL regeneration and rule out late-onset replacement resorption (ankylosis). The incisal tips of the intruded central incisors became visible in the oral cavity within the first week (Figure 3). By the end of four weeks, half of the crowns were clinically visible (Figure 4). Subsequent clinical assessments demonstrated that both incisors responded positively to cold testing and/or electric pulp testing (EPT), with no clinical signs of discoloration or sinus tracts. Furthermore, periapical radiographs revealed no periapical radiolucency, external inflammatory resorption, loss of the PDL space, or ankylosis-related changes. During the eruptive phase, continued vertical tooth movement was observed at the three-month follow-up, with no clinical or radiographic complications, such as tooth discoloration, sensitivity, or inflammatory root resorption (Figure 5).

**Figure 3:**
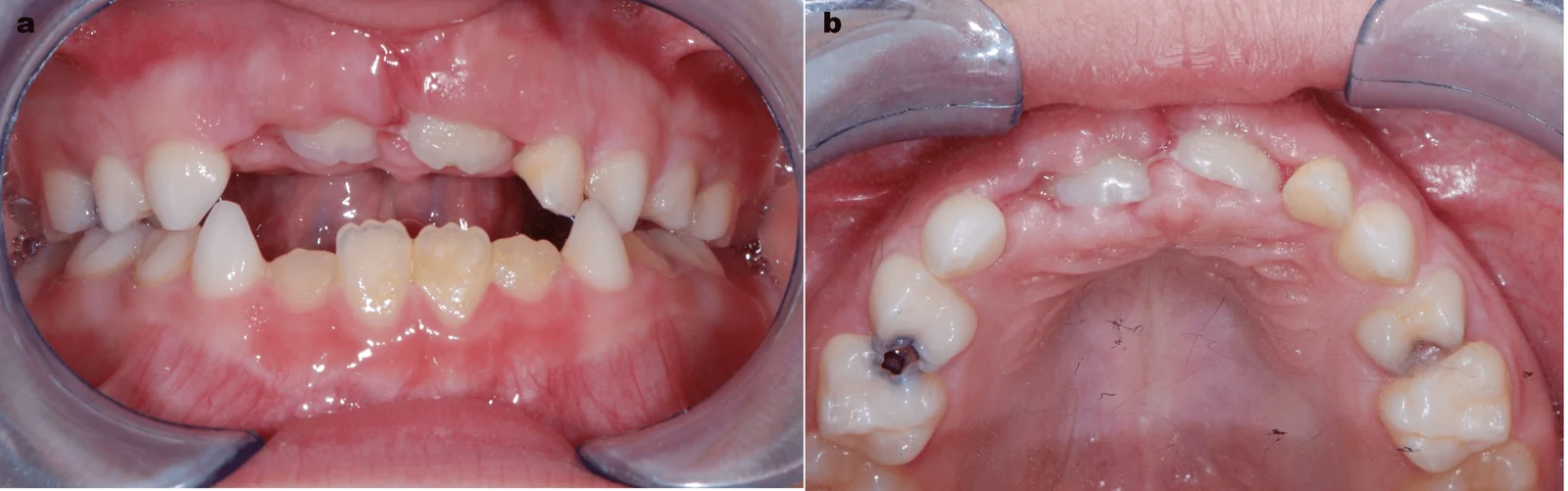
One-week follow-up: intraoral (a) frontal and (b) occlusal views demonstrating early spontaneous re-eruption, with the incisal tips of the intruded central incisors becoming visible in the oral cavity

**Figure 4:**
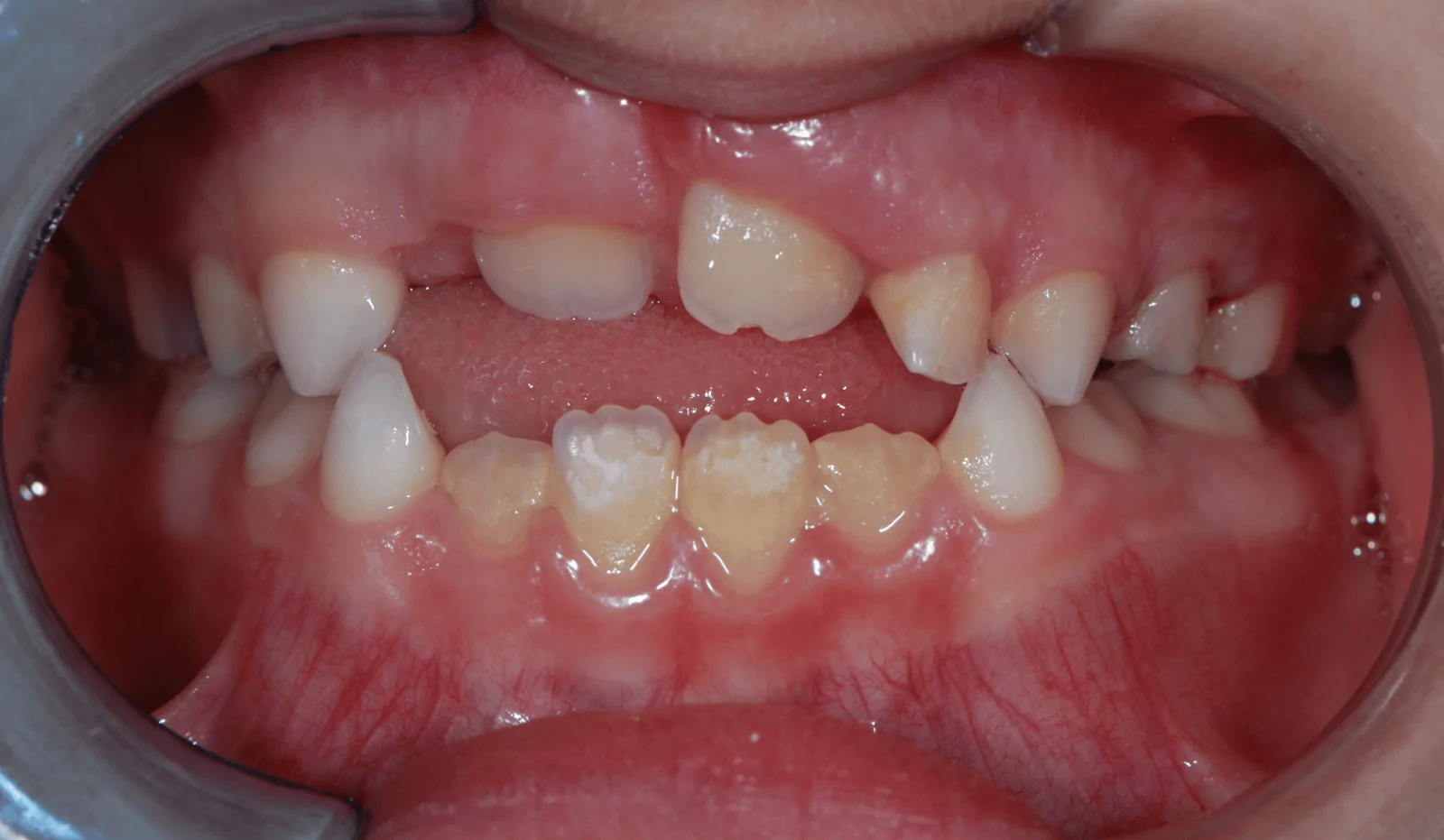
Four-week follow-up: intraoral frontal view showing continued spontaneous re-eruption, with approximately half of the clinical crowns visible

**Figure 5:**
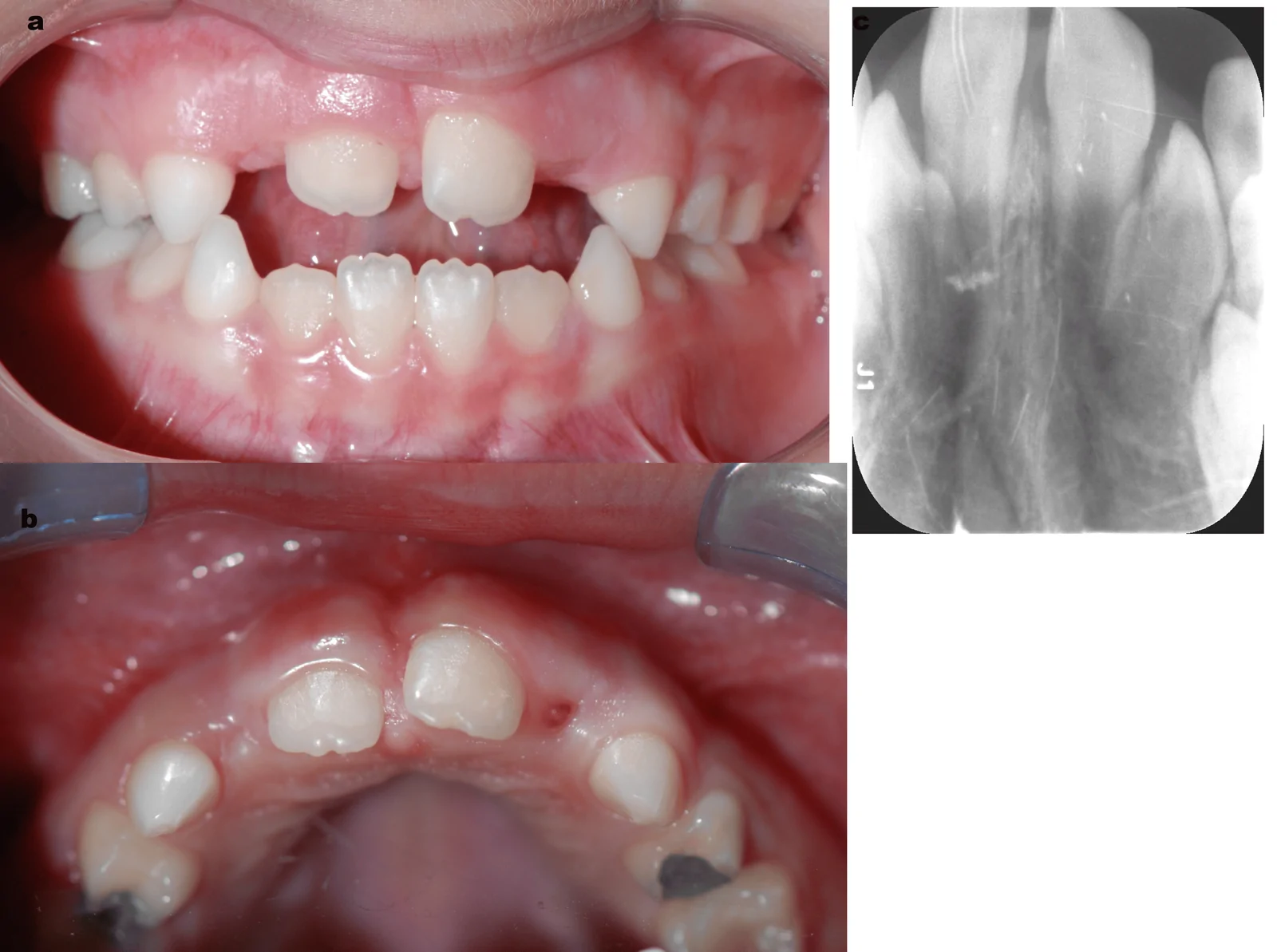
Three-month follow-up: intraoral (a) frontal and (b) occlusal views demonstrating progressive vertical repositioning of the central incisors. (c) Periapical radiograph confirming the absence of external inflammatory root resorption, periapical radiolucency, or ankylosis-related changes during the active eruptive phase

At the 14-month follow-up, the central incisors had fully erupted in the oral cavity, with continued root maturation. However, the right maxillary central incisor was more palatally displaced (retrusive) than the left central incisor (Figure 6). Although immediate orthodontic correction might be considered, a deliberate observational approach was maintained for two critical biological and biomechanical reasons: first, the application of active orthodontic forces to a recently re-erupted tooth with an immature, actively forming root significantly increases the risk of arresting root development and inducing external inflammatory root resorption. Second, the patient’s transitional mixed dentition phase posed a biomechanical challenge, and adequate stable posterior anchorage for a removable appliance was lacking owing to the physiological exfoliation of primary molars. Therefore, active orthodontic intervention was intentionally delayed to allow complete stabilization of the regenerated PDL, further apical maturation, and the establishment of reliable posterior anchorage. During the extended monitoring period, the teeth remained asymptomatic, with no clinical or radiographic complications.

**Figure 6:**
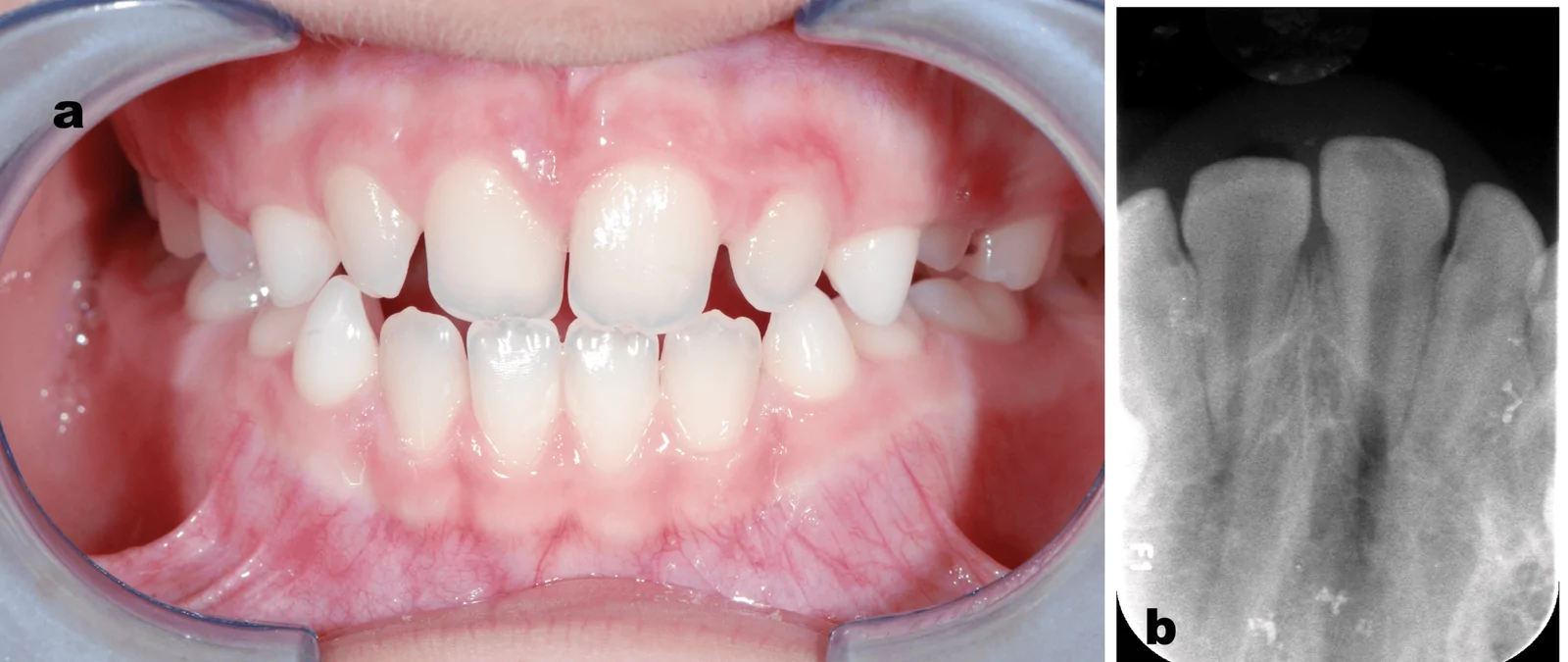
Fourteen-month follow-up: (a) intraoral frontal view showing complete eruption of both central incisors into the oral cavity. (b) Periapical radiograph confirming the continued absence of external inflammatory root resorption, periapical radiolucency, or ankylosis-related changes

At the 36-month follow-up, the teeth remained completely asymptomatic, with no clinical or radiographic complications (Figure 7). Once these optimal biological and biomechanical conditions were achieved, orthodontic intervention was initiated using a removable Hawley appliance with a protrusion screw (Figure 8). Orthodontic correction of the palatally displaced right maxillary central incisor was initiated using a custom-fabricated removable Hawley appliance incorporating an anterior protrusion screw positioned directly lingual to the target tooth, Adams clasps on the posterior molars for retention, and a passive labial bow (Figure 8). The patient's guardian was instructed to activate the protrusion screw by 0.25 mm (one-quarter turn) once per week. Full-time daily wear (22-24 hours/day, removed only during meals and toothbrushing) was prescribed. Active orthodontic protrusion was maintained for four months, successfully advancing the tooth into the desired overjet and alignment (Figure 9). Clinical examinations were conducted biweekly to monitor appliance seating and activation, while monthly periapical radiographs were obtained to confirm PDL space integrity, root maturation, and the absence of inflammatory root resorption. Following active movement, the appliance was rendered passive and worn as a nighttime retainer for six months to ensure long-term stability. After removable appliance treatment, the right central incisor was successfully advanced to the desired alignment, matching the adjacent teeth. At the end of treatment, during regular monitoring, both teeth had completed their alignment (Figure 9). Over a 58-month follow-up period, no signs of pulp necrosis or other clinical or radiographic complications, such as replacement root resorption (ankylosis) or pulp necrosis, were detected (Figure 10).

**Figure 7:**
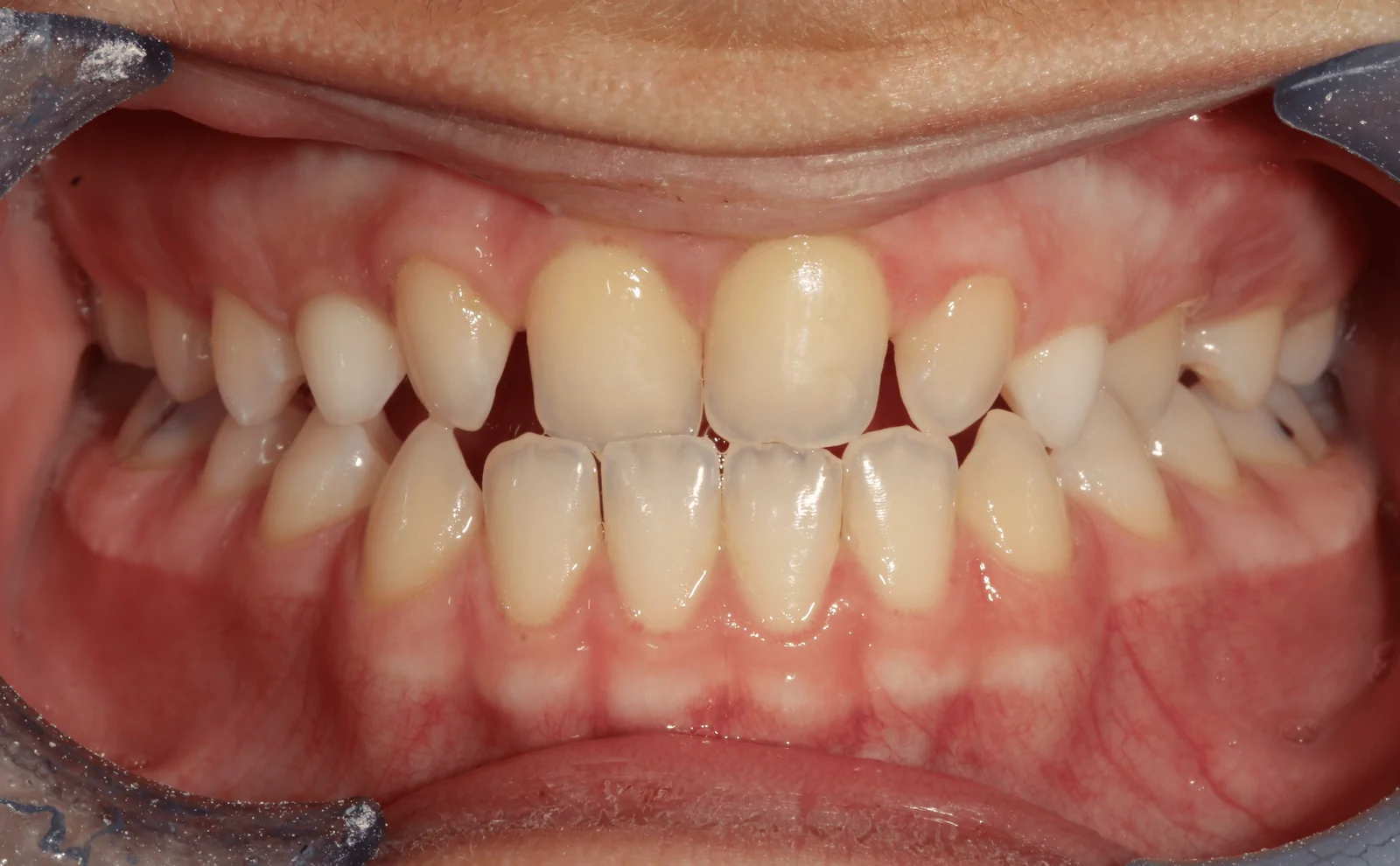
Thirty-six-month follow-up (before orthodontic intervention): intraoral occlusal view showing the maxillary right central incisor in a palatally displaced (retrusive) position relative to the left central incisor

**Figure 8:**
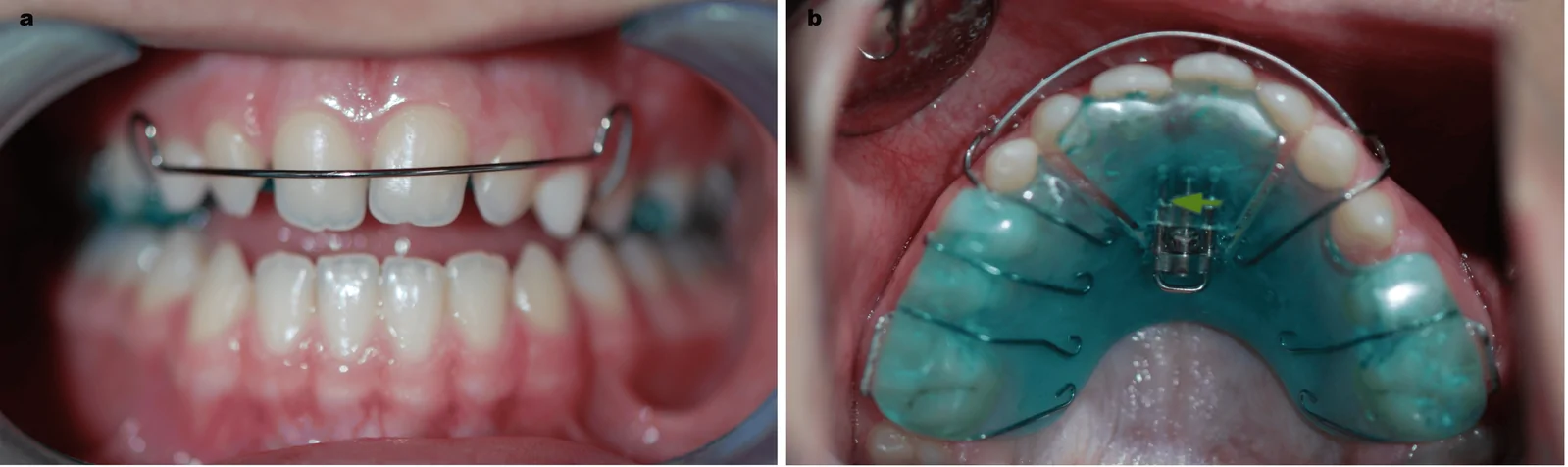
Active orthodontic intervention: intraoral (a) frontal and (b) occlusal views showing a removable Hawley appliance with a protrusion screw in place

**Figure 9:**
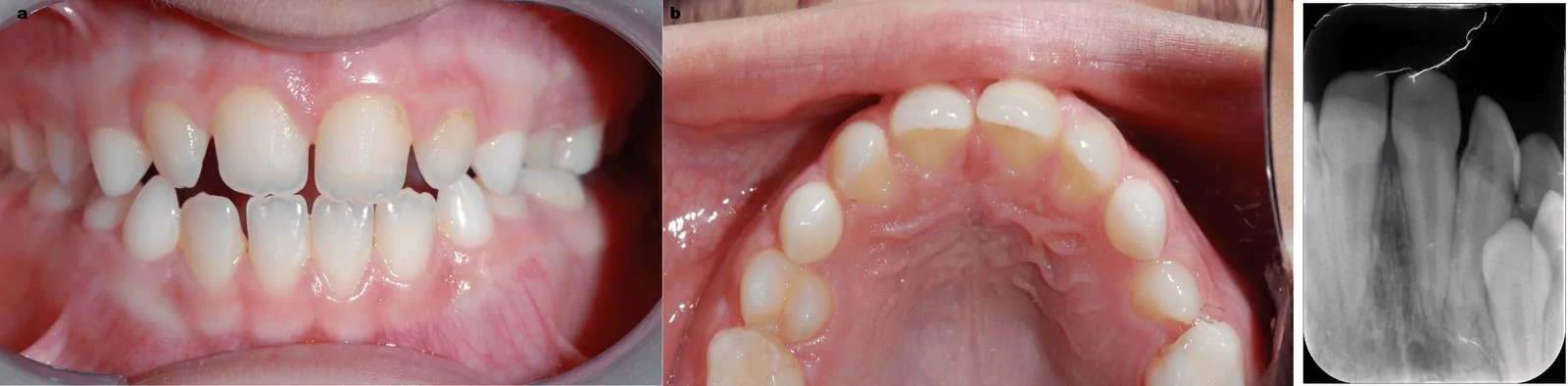
Forty-month follow-up: intraoral (a) frontal and (b) occlusal views demonstrating successful orthodontic alignment of the maxillary right central incisor. (c) Periapical radiograph confirming periodontal healing, absence of replacement root resorption, and preservation of pulpal health

**Figure 10:**
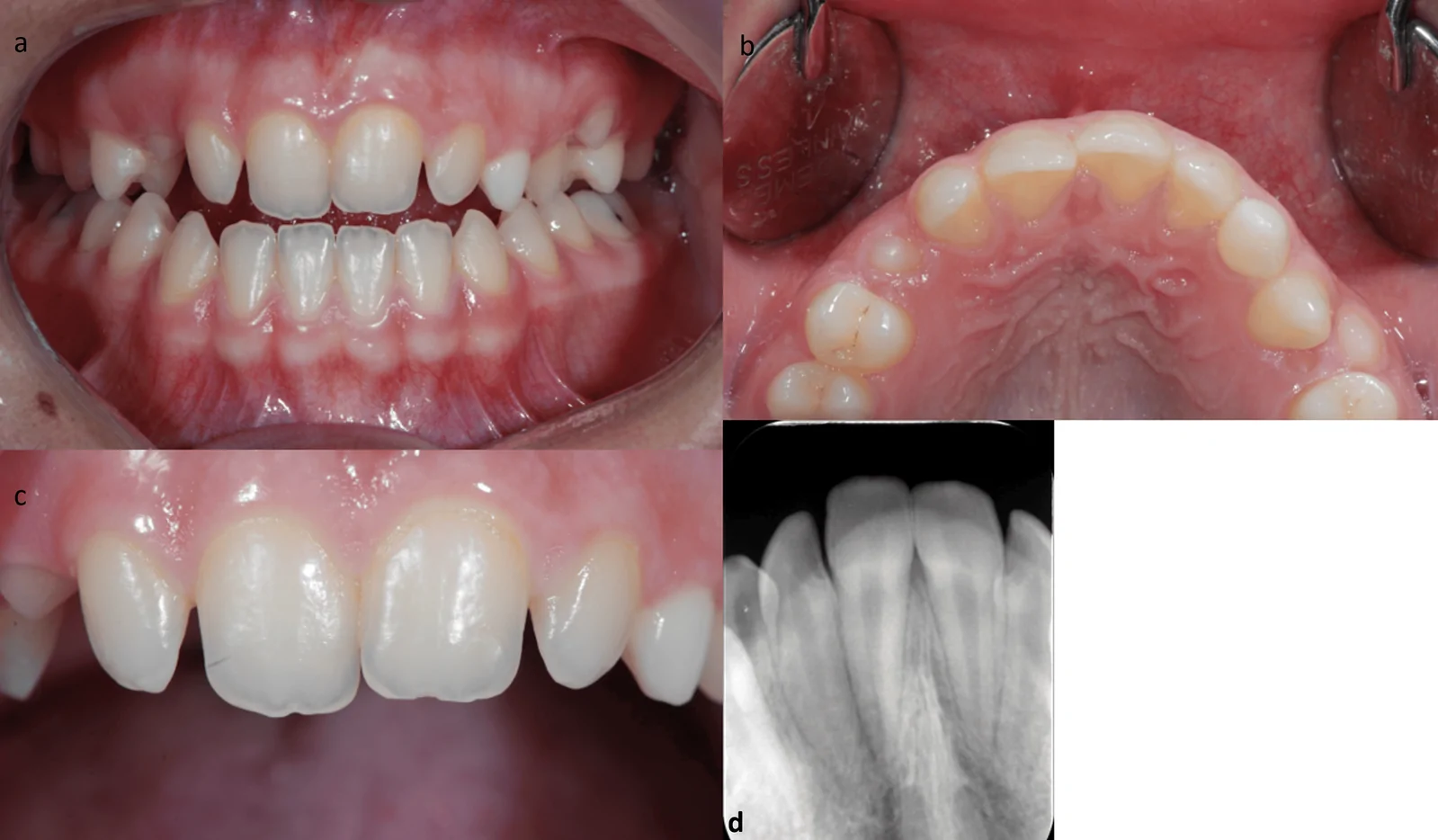
Fifty-eight-month follow-up: intraoral (a) frontal, (b) occlusal, and (c) macro views demonstrating orthodontic alignment and satisfactory aesthetics. (d) Periapical radiograph confirming functional periodontal healing and the absence of replacement root resorption

## Discussion

The management of severe intrusive luxation is highly complex because of the extensive crushing of the PDL, alveolar bone, and neurovascular supply [2]. In immature teeth, spontaneous re-eruption is the recommended first-line treatment according to IADT guidelines. This approach relies on the presence of a vital Hertwig’s epithelial root sheath and an open apex, which support continued root formation and periodontal healing [15]. Nevertheless, the choice between orthodontic extrusion, surgical repositioning, and endodontic treatment remains a subject of debate, particularly in cases of severe intrusive luxation [9].

Retrospective data consistently suggest that spontaneous re-eruption is highly probable in cases of mild-to-moderate intrusion. In contrast, it is rarely observed in severe intrusions (>7 mm) within the first few weeks because of a transient “locking mechanism” deep within the alveolar bone [5,17]. However, Singhal et al. demonstrated that teeth with root development at Cvek Stages 1 and 2 have significantly greater re-eruption potential and fewer complications than those at Stages 3 and 4 [16]. Consistent with these findings, the successful outcome in our patient underscores the eruptive potential of extremely immature teeth. Despite the generally poor prognosis associated with severe displacement, our case demonstrates that watchful waiting can result in complete spontaneous re-eruption within approximately 14 months of follow-up.

Patient age is a critical determinant of spontaneous re-eruption after an intrusive luxation injury. Children under nine years of age, such as our eight-year-old patient, may exhibit higher rates of re-eruption due to lower alveolar bone density and increased tissue vascularity. In the present case, the patient’s young age, combined with the early stage of root development (Cvek Stage 1), provided a favorable biological environment for healing, allowing the teeth to erupt within a timeframe consistent with the data reported by Wang et al. (0.5-18 months) [7] and Wigen et al. (3-12 months) [18].

Long-term follow-up is essential in intrusive luxation, as severe complications such as ankylosis (replacement root resorption) often become evident within the first few years after the injury [14]. Schweiger et al. and Tsilingaridis et al. noted that most ankylosis diagnoses occur within the first one to two years after trauma [5,19]. Severely intruded teeth also have a high incidence of pulp necrosis. Chacko and Pradhan [15] demonstrated that even within a two-week observation period for a 6-mm intrusion, external root resorption and pulp necrosis were present, necessitating a palatal flap for endodontic treatment. Furthermore, Snell et al. [20] reported a 10 mm intrusion with an uncomplicated crown fracture that required five months of preliminary orthodontic extrusion before endodontic intervention. In the present case, the favorable pulpal outcome may be explained by the wide-open apex (Cvek Stage 1), which theoretically facilitates revascularization, and the absence of concurrent crown fractures that could allow bacterial ingress into the ischemic pulp, along with other factors, including patient age, systemic antibiotic use, and individual healing capacity [21,22].

Post-eruption malpositions, such as the tipping of the maxillary central incisors observed in this case, are common consequences of the traumatic impact vector and altered eruption path during re-eruption after TDIs. Careful orthodontic intervention is required to correct these malpositions. As highlighted by Andreasen et al. [4], the application of early active orthodontic forces can inflict further trauma on already damaged periodontal tissues. In a recently re-erupted tooth with an immature, actively forming root, this significantly increases the risk of halting root development and inducing inflammatory root resorption. Although fixed continuous arch mechanics are conventionally used for alignment, their application during the mixed dentition stage is often biomechanically challenging because of the lack of suitable posterior anchorage units [23]. To overcome this anchorage limitation and avoid overloading adjacent developing teeth, a removable appliance with a protrusion screw was utilized in our case as a safe and viable alternative for isolated tipping corrections. Successful orthodontic movement of the previously intruded tooth indicates preserved periodontal function and confirms the absence of late-onset ankylosis during the 58-month follow-up. The radiographic and clinical findings were consistent with preserved periodontal function and healthy periodontal architecture.

The case report has some limitations. In young, developing teeth with open apices (Cvek Stage 1), the subodontoblastic neural plexus of Raschkow is developmentally incomplete, rendering sensory nerve responses (thermal and EPT) notoriously unreliable and prone to false-negative results. Furthermore, sensory tests evaluate neural function rather than pulpal blood perfusion. In the absence of advanced non-invasive vascular diagnostic tools such as laser Doppler flowmetry (LDF) or pulse oximetry, pulpal vitality and revascularization in our case were primarily confirmed indirectly through longitudinal radiographic evidence of continued apical root maturation, root dentin wall thickening, and the total absence of periapical radiolucency or root resorption.

## Conclusions

This case report supports conservative management as a viable first-line approach for severely intruded immature permanent teeth at an early stage of root development. However, as a single-case report, these findings cannot be generalized, and the relative contributions of patient age, root stage, injury severity, and individual healing capacity remain uncontrolled. Larger prospective studies are needed to validate the prognostic utility of Cvek’s classification and establish evidence-based treatment protocols for severe intrusion.
